# 

**Published:** 1913-06-21

**Authors:** 


### Cancer Research in Denmark.

What may turn out to be a research of tremendous importance has been published lately by Professor Fibiger, of Copenhagen, in the *Berliner Klinische Wochenschrift*. This observer a long time ago discovered in some rats certain papillomatous-looking tumours of the stomach which proved, on miscroscopical examination, to be cancerous, and also to contain in their interior parasitic worms of the nematode genus. For some time he was unsuccessful in the search for further material of the same sort to work on, but at last he came across the same condition in rats from a sugar refinery. Two-thirds of these animals harboured the nematode worms, and nine out of forty had also the peculiar stomach growth in association therewith. Fibiger tried by artificially infecting rats with the worms to reproduce the stomach cancer, but failed. Thinking that this might indicate an intermediate host, he hit upon the cockroach, which insect existed in large numbers at the sugar works. By feeding rats on these cockroaches he produced thirty-six cases of this particular stomach-tumour out of fifty-seven rats. In a few of the cases secondary growths also appeared, and these did not contain nematodes. The hypothesis of an intermediate host was further proved by feeding cockroaches on the fæces of the rats wherein the ova of the worms are contained. It is still matter for speculation whether these observations prove that any irritant may set up cancerous tissue changes; or whether they point to a specific cancer parasite. If the latter, further research with this clue to help should result in its discovery.

### The Use of Sumbul.

A drug which is not very often prescribed nowadays in England is sumbul root, of which the tincture is officinal in the British Pharmacopœia. Its chief action, according to Hale White, is as a carminative; and, as it also contains valerianic acid, it has been used a good deal for neurasthenia and functional nervous disorders. Sumbul is contained in one, if not more, of the secret quack medicines which are under inquisition at the hands of a Parliamentary Commission. It was formerly a favourite for combination with bromides, though one eminent authority in London who thus prescribed it avowed openly that he did so merely because it very effectively covered the nasty taste of bromine salts. Dr. Macht has been investigating sumbul clinically at the Johns Hopkins Hospital, and he has come to the conclusion that it is quite useless for any and every kind of functional nervous disorder. In a small percentage of cases some temporary improvement followed its exhibition; but there was no lasting benefit.

### Buildings Contemplated and in Progress.

DURHAM.—A committee has been appointed by the Guardians to select a site for a new hospital.

EDMONTON, ALBERTA.—It is estimated that the civic hospital which it is proposed to build on the University ground at Edmonton, Alberta, involves an expenditure of £43,000.

FORD.—The Local Government Board has given sanction to the Sunderland Rural District Council to borrow the sum of £2,630 from the Public Works Loan Commissioners for the purchase of land and for additions to the isolation hospital.

GOOLE.—The Local Government Board has authorised the Guardians to expend a sum not exceeding £4,500 upon alterations and additions to the workhouse infirmary.

HASTINGS.—The offer of the Hastings Corporation to purchase the site of the East Sussex Hospital for £15,050 has been accepted by the hospital committee. It is hoped that the building of the new hospital will be commenced at an early date.

MIDDLESBROUGH.—The Local Government Board has sanctioned a loan of £250 towards the cost of purchasing premises for a dispensary.

NEWPORT, MON.—As an addition to the Newport Hospital, Lord Tredegar has given a large mansion and land, valued at £20,000.

RUTHIN.—Amended plans have been approved by the Guardians for an infirmary estimated to cost £3,500.

SKEGNESS.—The new Skegness and District Cottage Hospital, built at a cost of £1,450, has been formally opened. The architect was Mr. Parkinson, of Blackburn.

TREDEGAR.—The Urban District Council have passed plans for extensions to the Cottage Hospital.

### A CURIOUS DONATION.

BY an odd coincidence the Research Defence Society has received £30 as a donation, which sum was paid by one of the Anti-Vivisection Societies for a month's tenancy of some Kensington premises. It appears that the owner, finding to what use the premises are put, has given the rent derived from the letting of them to the Research Defence Society.

### Appointments.

Mr. Cecil Rowntree, M.B., B.S., F.R.C.S., has been appointed assistant surgeon to the *Dreadnought* Hospital.

Dr. Herbert French, F.R.C.P. (Lond.), has been appointed examiner in medicine to the Society of Apothecaries, London.

Dr. Stanley D. Craig, M.B., Ch.B., has been appointed house surgeon at the Stockton and Thornaby Hospital. He has been senior house physician at the West Ham and Eastern General Hospital.

Dr. James H. Martin has been appointed obstetric physician to the West End Branch of the Glasgow Maternity and Women's Hospital. Dr. Charles Brash and Dr. A. Bruce Maclean have been appointed outdoor house surgeons at the hospital for three months from July 5, and Dr. Mary Van Ingen has been appointed outdoor house surgeon at the West End Branch for the same period.

### Personalia.

Sir Henry Lopes, Bart., has been elected a vice-president of the Royal Hospital for Incurables, Putney Heath.

Her Highness Princess Marie Louise of Schleswig-Holstein has become patron of the Kensington and Fulham General Hospital.

Lord Massereene and Ferrard has been appointed a member of the Manor Mental Hospital Sub-Committee of the London County Council.

The Camberwell Board of Guardians have consented to close their dispensaries on Saturdays at two o'clock, in order to ease their dispensers' hours. Urgent requirements will be dealt with by local chemists in business.

The King has been graciously pleased to appoint Colonel William Kinnear, M.D., Assistant Director of Medical Services of the Highland Territorial Division, and Colonel Charles Pye Oliver, M.D., Assistant Director of Medical Services of the Home Counties Territorial Division, to be Honorary Physicians to his Majesty.

Mr. F. H. Roby has been elected vice-chairman of the board of governors of Manchester Children's Hospital, Pendlebury, and chairman of the dispensary committee; and Mr. Alfred K. Armitage, J.P., chairman of the house committee. Mr. Walter B. Dendy has been elected chairman of the convalescent branch committee.

Manchester and District Association of Hospital Secretaries.—By kind invitation of Mr. William P. Park, J.P., chairman, the members of this Association paid a visit to the Royal Infirmary, Preston, and were shown over the infirmary by the Chairman and Miss Pritchard, assistant matron. The next quarterly visit takes place in September, when the Liverpool Royal Infirmary will be visited.

### THE RICHARD MURRAY HOSPITAL.

Mr. Richard Murray, of Harrogate, who left estate, "as far as at present can be ascertained," of the gross value of £558,263, of which the net personalty has been sworn at £509,966, confirmed the recent gift by him of his Benfieldside and Tinkler Hall estates, £10,000, 2,000 £5 Preference shares, and £10,000 Debentures of the North-Eastern Breweries, Ltd., to trustees for the erection and endowment of the "Richard Murray Hospital."

### York County Hospital.

SHALL THERE BE A THOROUGH REORGANISATION OR THE PERPETUATION OF THE OLD WANT OF SYSTEM?

It is reported that at the meeting of the House Committee of the York County Hospital, held at the hospital on the 13th instant, the Dean of York in the chair, the report on the recent hospital inquiry came up for discussion, and a sub-committee was appointed to go into the whole question and report to a meeting of the House Committee to be subsequently held. The House Committee, being in fact mainly responsible for the executive and internal management of the hospital during the period to which the inquiry related, are closely allied to the abuses complained of, and it is held by independent governors and the outside world that the sub-committee would be wise to recommend the appointment of a small representative committee of outside governors of influence to deal with the Commissioners' report and to recommend the necessary changes and reforms.

### Gifts and Bequests.

The Chelsea Hospital for Women has received £26 5s. from the Mercers' Company.

The Maternity Charity and District Nurses' Home, Plaistow, E., has received £20 from Lord Portman.

Mr. Herbert Winstanley, architect, has bequeathed £500 to the Royal Female Orphan Asylum, Beddington.

The South London Hospital for Women has received towards the building fund £100 from the Clothworkers' Company.

Mr. Henry Gibson Anderson has left £1,500 to the Arbroath Infirmary and £500 to the British Hospital at Montevideo.

A legacy of £1,000 has been bequeathed to the Throat Hospital, Golden Square, by the late Mrs. F. G. Smart, of Tunbridge Wells.

### Obituary.

Dr. William Henry Langley, C.M.G., principal medical officer in Southern Nigeria since 1911, has died. Educated at Dublin, and assistant surgeon at Richmond Hospital, Dublin, he entered the Colonial Medical Service. For his services with the Kano-Sokoto expedition of 1903 he received the C.M.G. His Colonial posts included those of principal medical officer on the Gold Coast Colony, and deputy principal medical officer in Northern Nigeria.

Dr. Nathaniel Henry Alcock, Professor of Physiology at McGill University, has died at Montreal, aged forty-two. Educated at Dublin, London, and Marburg, he became senior moderator and gold medallist at Dublin in 1896, and afterwards held appointments at Victoria University, Manchester, and Dublin, and then became lecturer on physiology at St. Mary's Hospital Medical School, London. He had been appointed to his professorship at McGill University in 1911.

### DOCTORS' WILLS.

Sir Henry Rosborough Swanzy, M.D., of Dublin, who died on April 12, aged seventy, left personal estate in the United Kingdom valued at £12,487.

